# Efficient targeted mutagenesis of rice and tobacco genomes using Cpf1 from *Francisella novicida*

**DOI:** 10.1038/srep38169

**Published:** 2016-12-01

**Authors:** Akira Endo, Mikami Masafumi, Hidetaka Kaya, Seiichi Toki

**Affiliations:** 1 Plant Genome Engineering Research Unit, Institute of Agrobiological Sciences, National Agriculture and Food Research Organization, 2-1-2 Kannondai, Tsukuba, Ibaraki 305-8602, Japan; 2 Graduate School of Nanobioscience, Yokohama City University, 22-2 Seto, Yokohama, Kanagawa 236-0027, Japan; 3 Kihara Institute for Biological Research, Yokohama City University, 641-12 Maioka-cho, Yokohama, Kanagawa 244-0813, Japan

## Abstract

CRISPR/Cas9 systems are nowadays applied extensively to effect genome editing in various organisms including plants. CRISPR from *Prevotella* and *Francisella* 1 (Cpf1) is a newly characterized RNA-guided endonuclease that has two distinct features as compared to Cas9. First, Cpf1 utilizes a thymidine-rich protospacer adjacent motif (PAM) while Cas9 prefers a guanidine-rich PAM. Cpf1 could be used as a sequence-specific nuclease to target AT-rich regions of a genome that Cas9 had difficulty accessing. Second, Cpf1 generates DNA ends with a 5′ overhang, whereas Cas9 creates blunt DNA ends after cleavage. “Sticky” DNA ends should increase the efficiency of insertion of a desired DNA fragment into the Cpf1-cleaved site using complementary DNA ends. Therefore, Cpf1 could be a potent tool for precise genome engineering. To evaluate whether Cpf1 can be applied to plant genome editing, we selected Cpf1 from *Francisella novicida* (FnCpf1), which recognizes a shorter PAM (TTN) within known Cpf1 proteins, and applied it to targeted mutagenesis in tobacco and rice. Our results show that targeted mutagenesis had occurred in transgenic plants expressing FnCpf1 with crRNA. Deletions of the targeted region were the most frequently observed mutations. Our results demonstrate that FnCpf1 can be applied successfully to genome engineering in plants.

Targeted mutagenesis and gene targeting using sequence specific nucleases (SSNs) are powerful strategies used to accelerate molecular breeding of crops. Several types of SSN, such as ZFNs (zinc-finger nucleases), TALENs (transcription-activator-like effector nucleases) and CRISPR/Cas9 (clustered regularly interspaced short palindromic repeats/CRISPR-associated protein 9) have been intensively adapted for use in genome editing in plants^1^,^2^,^3^. The number of papers reporting plant genome engineering with CRISPR/Cas9 has increased markedly during the last few years^4^, highlighting the fact that CRISPR/Cas9 is a versatile tool with which to perform targeted mutagenesis in plants^5^.

Several types of CRISPR/Cas9 systems are known to function as adaptive immune systems in archaea and bacteria^6^. The well-characterized CRISPR/Cas9 system is categorized as a class 2/type II immune system comprised of single-component effector proteins, and has been engineered for genome editing^7^,^8^. Cas9 protein is an endonuclease functioning with CRISPR RNA (crRNA) and transactivating crRNA (tracr RNA)^9^. The Cas9 RNA complex scans double-stranded DNA to find a DNA sequence complementary to the 20-nucleotide (nt) spacer region (target sequence) within the crRNA, as well as a protospacer adjacent motif (PAM), and then cleaves the target sequence on the invader DNA^9^. The recognition sequence of the PAM, which is located immediately downstream of the target sequence, varies in each Cas9 protein^6^,^7^,^10^. Widely used Cas9 proteins from *Streptococcus pyogenes* (SpCas9) and *Staphylococcus aureus* (SaCas9) prefer a guanidine-rich PAM, with the PAM sequences of SpCas9 and SaCas9 being NGG and NGRRT, respectively^6^,^11^,^12^.

Recently, Cpf1–a new type of RNA-directed endonuclease—was classified as class 2/type V in the CRISPR/Cas system^8^,^13^. Some features of Cpf1 differ from those of Cas9 although their functions are similar. While the Cas9 RNA complex contains two RNA molecules in nature, Cpf1 functions with a single crRNA to search and cleave target sequences in infiltrator DNA. A PAM sequence, located immediately upstream of the spacer sequence (target sequence), is also necessary for recognition of the 24 nt target sequence of Cpf1^13^. PAM recognition sequences of Cpf1 are different among bacterial species, and known Cpf1 proteins tend to utilize a thymidine-rich PAM^13^. The PAM sequence of Cpf1 from *Francisella novicida* is TTN. TTTN is recognized as PAM by Cpf1 isolated from *Acidaminococcus* sp. BV3L6 (AsCpf1) and *Lachnospiraceae bacterium* MA2020 (LbCpf1)^13^. In addition, Cpf1 and Cas9 generate different types of DNA ends after cleavage of the target sequence. Cpf1 creates DNA ends with a 5′ overhang, while Cas9 generates blunt ends^13^. Cleavage by FnCpf1 occurs at the 18^th^ base from PAM on the non-targeted (+) strand, and at the 23rd base from PAM on the targeted (–) strand within 24 nt spacer sequence^13^. Since DSBs with compatible overhangs can be repaired via precise end joining^14^, the sticky DNA ends generated by Cpf1 are thought to be ideally suited to precise genome editing such as knock-in or replacement of a desired DNA fragment using compatible DNA ends. These specific features of Cpf1 can broaden the spectrum of genome editing that is possible using SSNs.

To apply Cpf1 to plant genome engineering, we selected FnCpf1 for the following reasons. Since FnCpf1 recognizes TTN as PAM sequence, the frequency of target sequences for FnCpf1 in plant genomes is thought to be higher than that of AsCpf1 and LbCpf1, which utilize TTTN as a PAM sequence^13^. The shorter PAM of FnCpf1 is thus a practical and favorable feature for targeted mutagenesis, although the genome editing activity of FnCpf1 in human cells is reported to be lower than that of AsCpf1 and LbCpf1^13^. Targeted mutagenesis of plants has been performed mostly via stable transformation, introducing T-DNA harboring SSNs into plant genomes^15^,^16^. We hypothesized that the lower genome editing activity of FnCpf1 might be compensated by the constitutive expression of FnCpf1 in plants. We first engineered a binary vector to optimize the expression of FnCpf1 in plants, then designed a targeted mutagenesis experiment in tobacco and rice.

## Results

### FnCpf1 expression vectors for targeted mutagenesis in tobacco and rice

**Figure 1:**
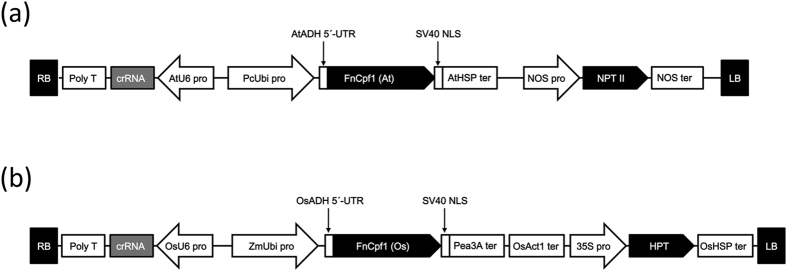
T-DNA constructions for FnCpf1 expression in tobacco and rice. (**a**) Construct for targeted mutagenesis in tobacco. FnCpf1 (At) was inserted downstream of the *PcUbi* promoter. The *Athsp* terminator was placed at the end of *FnCpf1* ORF. The *AtADH* 5′-UTR was introduced between the *PcUbi* promoter and FnCpf1 (At) to enhance translation. the nuclear localization signal (NLS) from the SV40 large T-antigen was fused translationally to the C-terminus of FnCpf1. The crRNA is under the control of Arabidopsis U6-26 promoter. To isolate transformants with kanamycin resistance, an *NPT II* cassette was included in the construct. *AtADH* 5′-UTR: 5′ untranslated region of *Arabidopsis thaliana ALCOHOLDEHYDROGENASE* gene. *Athsp* ter: the terminator region of *Arabidopsis thaliana HEAT SHOCK PROTEIN 18.2* gene. (**b**) Construct used for targeted mutagenesis in rice. The *ZmUbi-1* promoter drives expression of FnCpf1 (Os). The *OsADH* 5′-UTR was introduced between the ZmUbi promoter and FnCpf1 (At) to enhance translation. An NLS was fused translationally to the C-terminus of FnCpf1 (Os). Pea3A and OsAct1 terminators were inserted tandemly downstream of FnCpf1 to terminate transcription. Expression of crRNA is driven by the rice U6-2 promoter. To screen transformants with hygromycin resistance, HPT cassettes were included in the construct. *OsADH* 5′-UTR: 5′ untranslated region of *Oryza sativa ALCOHOLDEHYDROGENASE* gene. *Pea3A* ter: the terminator region of *Pisum sativum rbcS 3A* gene. OsAct ter: the terminator region of *Oryza sativa Actin* gene.

To perform targeted mutagenesis using FnCpf1 in tobacco and rice, we first constructed binary vectors harboring *FnCpf1* and antibiotic resistance genes in the T-DNA region (Fig. 1a,b) (cf. our previous construction of binary vectors to express SpCas9^12^,^17^). The codon usage of *FnCpf1* ORF was optimized for effective translation in *A. thaliana* and rice, respectively. Codon-optimized *FnCpf1* (At) and *FnCpf1* (Os) were cloned into the binary vectors, pRI201-AN and pPZP200, respectively. FnCpf1 (At) was driven by the ubiquitin 4–2 promoter from *Petroselinum crispum* (Fig. 1a)^18^,^19^. On the other hand, FnCpf1 (Os) was placed under the control of the ubiqcruitin promoter from *Zea mays* (Fig. 1b)^20^. To express crRNA of FnCpf1, Arabidopsis U6-26 and rice U6-2 small nuclear RNA gene promoters were used in tobacco and rice, respectively (Fig. 1a,b)^17^,^19^.

### Targeted mutagenesis in tobacco

To examine whether FnCpf1 (At) can induce targeted mutation in tobacco, 24 nt target sequences were designed to induce mutations in two genes, i.e., *phytoene desaturase (NtPDS*) and *STENOFOLIA* ortholog in *Nicotiana tabacum (NtSTF1*). Mutation in *NtPDS* will cause an albino phenotype since a defect in carotenoid biosynthesis leads to loss of pigments such as chlorophyll^21^. *NtSTF1* is thought to be involved in leaf blade expansion since a *lam* mutant having a defect in *LAM (NsSTF1*) shows a narrow leaf phenotype in *Nicotiana sylvestris*^22^,^23^*. Nicotiana tabacum* is an amphidiploid species derived from ancestors that are closely related to the diploid species *N. sylvestris* and *N. tomentosiformis*. Therefore, mutant phenotypes can be observed when mutations occur in functionally identical genes located in the *N. sylvestris* and *N. tomentosiformis* genomes (S and T genomes), respectively. To select target sequences in these genes on both S and T genomes, TTN, a PAM sequence of FnCpf1, is first searched for within exons of these two genes, and then, 24 nt sequences immediately downstream of the PAM were selected as target sequence. Two and four target sequences, respectively, were designed against *NtPDS* and *NtSTF1* genes (Table 1). These crRNAs were named as follows, cr*NtPDS*-1 and cr*NtPDS*-2, and cr*NtSTF1*-1–cr*NtSTF1*-4. The mutation ratio was estimated by scoring the number of regenerated plants with mutation around the target sequence of FnCpf1 relative to the total number of regenerated plants. The mutation frequency represented the ratio of mutated clones per total randomly sequenced clones.

**Figure 2:**
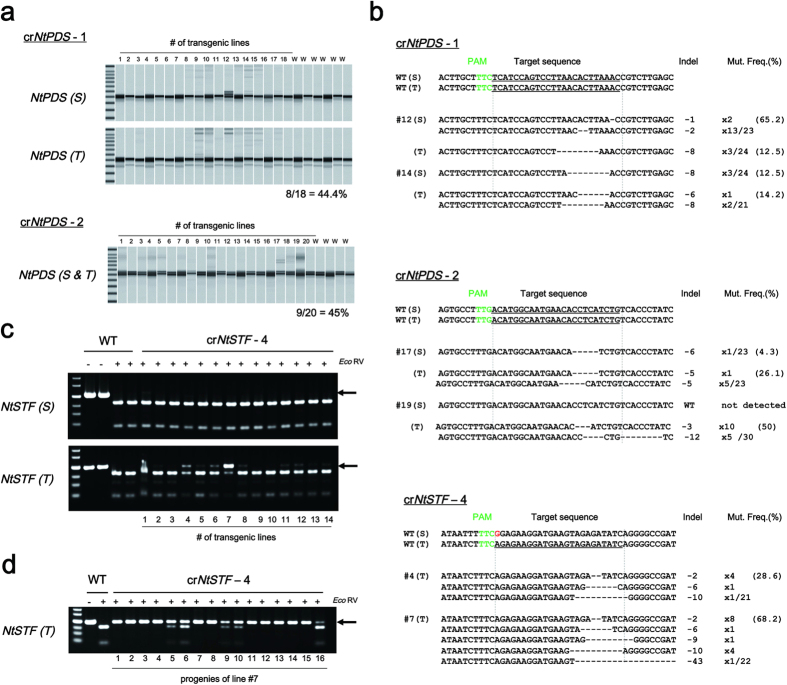
Analyses of FnCpf1-induced mutations in tobacco. (**a**) Heteroduplex mobility assay to detect mutation on cr*NtPDS*-1 (upper two panels) and cr*NtPDS*-2 (lower panel) loci. (S) and (T) indicate PCR products amplified from the loci including each target sequence on *N. sylvestris* and *N. tomentosiformis* genomes, respectively. (**b**) Patterns of mutations detected in cr*NtPDS*-1 (top), cr*NtPDS*-2 (middle) and cr*NtSTF*-4 (bottom) loci. The target DNA sequences of each crRNA are shown as wild-type (WT) at the top with underlined. (S) and (T) indicate the target sequence on both S and T genomes. The PAM regions are shown by green. Mismatched nucleotide is indicated in red. Line numbers of transgenic plants were indicated as # at left side of each sequence. DNA deletions are presented as dashes. The length of indel and the number of clones are represented at the right side of each sequence (+, insertion; −, deletion; ×, number of clones). Mut. Freq. (%): Mutation frequency. (**c**) CAPS analysis of cr*NtSTF*-4 locus in T0 generation. (**d**) CAPS analysis of cr*NtSTF*-4 locus in T1 generation of line #7. −: Non-digested PCR products, +: *Eco*RV-digested PCR products. Arrow head indicated the position of undigested PCR products. An undigested band indicates mutation at the cr*NtSTF*-4 locus.

Transgenic T0 plants showing kanamycin resistance (between 14 and 20 lines) were isolated for each of the target loci. Genomic DNA was isolated from each T0 transgenic plant, and the target loci were then amplified by PCR. To detect mutations in *NtPDS* genes, PCR products were resolved by performing a heteroduplex mobility assay (HMA). As shown in Fig. 2a, DNA bands with higher molecular weights were observed in several transgenic lines but not in wild-type (WT) (Fig. 2a). Mutation ratios at cr*NtPDS*-1 and cr*NtPDS*-2 loci were around 45%. Mutation patterns of these transgenic lines were analyzed by DNA sequencing, and mutation frequencies were estimated. Deletion mutations were observed around the cleavage site of FnCpf1 (Fig. 2b). Mutation frequencies at cr*NtPDS*-1 and cr*NtPDS*-2 loci were 12.5–65.2% and 4.3–50%, respectively (Fig. 2b, top and middle). Regardless of crRNA, mutation frequencies on the S and T genomes had no consistency in any of the transgenic plants tested (Fig. 2b, top and middle). FnCpf1-induced mutations on the S or T genome seem to occur stochastically at *NtPDS* loci.

Among the four crRNAs designed to target *NtSTF1*, cr*NtSTF1*-1 to cr*NtSTF1*-3 did not induce mutation in any of the transgenic lines (Supplemental Fig. 1), while mutations were seen with cr*NtSTF1*-4. This latter crRNA was able to target the *NtSTF1* gene in the *N. tomentosiformis* genome but not in the *N. sylvestris* genome since cr*NtSTF1*-4 had a one-base mismatched sequence against *NtSTF1 (S)* (Fig. 2b, lower). To find mutations in *NtSTF1*, PCR products were subjected to CAPS assay. The mutation ratios of cr*NtSTF1*-4 at the S and T loci were 7.1% and 71.4%, respectively (Fig. 2c). Mutation frequencies of cr*NtSTF1*-4 on the T locus were 28.6–68.2% (Fig. 2b, bottom). These results clearly showed that FnCpf1 could induce mutation at target sites in tobacco. However, we could not recover transgenic plants harboring biallelic mutations at the target sites in the T0 generation.

We next confirmed whether FnCpf1-induced mutations on cr*NtSTF1*-4 locus are genetically transmitted to the next generation. PCR was performed using DNA extracted from progenies of cr*NtSTF1*-t4 line #7 and used for CAPS analysis. As a result, homoallelic mutation was observed in some of the progenies from transgenic tobacco line #7 (Fig. 2d).

### Targeted mutagenesis in rice

Next, we applied FnCpf1 (Os) to induce mutation in rice. *Agrobacterium*-mediated transformation was performed to introduce the T-DNA harboring *FnCpf1 (Os)* into scutellum-derived calli. The genes *OsDrooping leaf (OsDL*) and *OsAcetolactone synthase (OsALS*) were selected as the target genes. *dl* mutants show a loss of midrib in the leaf blade, resulting in a drooping leaf phenotype^24^,^25^. Acetolactone synthase is involved in the synthesis of branched-chain amino acids^26^,^27^. Loss of ALS activity leads to lethality. As in tobacco, two target sequences were designed for targeted mutagenesis of each gene. The corresponding crRNAs were named cr*OsDL*-1~2, and cr*OsALS*-1~2 (Table 1). The digestion sites of both FnCpf1 and restriction enzymes were designed to overlap within the target sequences, so that the restriction site would be disrupted if mutations occur at the target loci following cleavage by FnCpf1. The CAPS assay was used to assess the presence of mutations in the target sequence.

**Figure 3:**
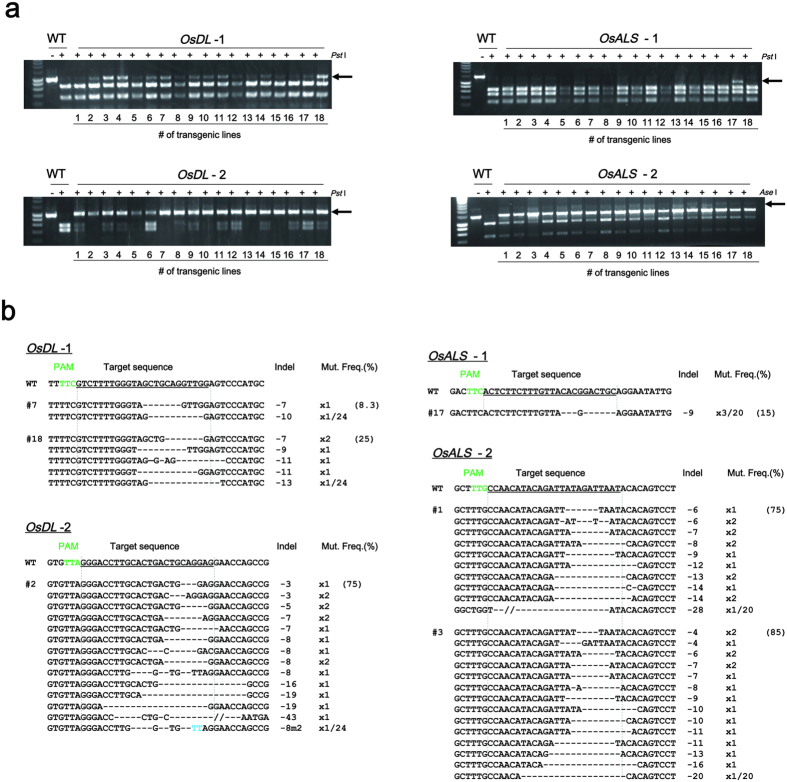
Analyses of FnCpf1-induced mutations in rice. (**a**) CAPS analysis of cr*OsDL*-1~2 and cr*OsALS*-1~2 loci. −: Non-digested PCR products, +: *Pst*I or *Ase*I-digested PCR products. Arrow head indicated the position of undigested PCR products. An undigested band indicates mutation in the target loci. (**b**) Patterns of mutations detected in cr*OsDL*-1~2 and cr*OsALS*-1~2 loci. The target DNA sequences of each crRNA are shown as wild-type (WT) at the top with underlined. (S) and (T) indicate the target sequence on both S and T genomes. The PAM regions are shown by green. Mismatched nucleotide is indicated in red. Line numbers of transgenic plants were indicated as # at left side of each sequence. DNA deletions are presented as dashes. The length of indel and the number of clones are represented at the right side of each sequence (+, insertion; −, deletion; ×, number of clones). Mut. Freq. (%): Mutation frequency.

To examine FnCpf1-induced mutations in rice calli, genomic DNA was extracted from each callus showing hygromycin resistance. Target loci were amplified by PCR, and the products were subjected to CAPS assay (Fig. 3a). Undigested PCR products were observed in transgenic lines, indicating that the introduction of *FnCpf1* with each of the crRNAs was able to induce mutations in rice calli. At the cr*OsDL*-2 and cr*OsALS*-2 target loci, the mutation frequency in rice calli was over 60% (Fig. 3b). Mutation frequencies at the cr*OsDL*-1 and cr*OsALS*-1 target loci were 8.3–25% and 15%, respectively (Fig. 3b). As shown in Fig. 3b, deletions occurred mostly at target sites of all crRNA. In the case of regenerated plants obtained from transgenic calli harboring FnCpf1 with cr*OsDL*-2 and cr*OsALS*-2, the mutation ratio of the regenerated plants at the cr*OsDL*-2 target locus was 85.7% (6/7), and the three plants with bi-allelic mutations showed a drooping leaf phenotype in the T0 generation (Supplemental Fig. 2). In addition, the ratio of regenerated plants with mutation at the cr*OsALS*-2 target locus was 90% (9/10), and five bi-allelic mutant plants were obtained. Because all the bi-allelic mutant plants had deletions with mutations that did not generate frameshifts on the *OsALS* gene, these *als* mutants were assumed to be viable (data not shown). In addition, heteroallelic mutation on cr*OsDL*-1 target locus was inherited as homoallelic mutation in the progeny of line #18 (Supplemental Fig. 3). These results clearly indicate that FnCpf1 is able to cleave target sites in rice.

### Off target mutation analysis of FnCpf1 in rice

Zetsche *et al*. reported that the seed region of the FnCpf1 crRNA is within the first 5 nt of the 5′-end of the spacer sequence *in vitro*^13^. We tried to examine the possibility of FnCpf1-induced mutations at off-target genes in rice. To explore this possibility, we selected the 9-*cis*-*epoxycarotenoid dioxygenase (NCED*) gene family (*OsNCED1*–*3*), and the *aldehyde oxidase (AO*) gene family (*OsAO1*–*5*)^28^,^29^. When the *crOsNCED1-1* sequence of the *OsNCED1* gene was defined as the target sequence, the corresponding sequence of the *OsNCED*2 has one, and that of OsNCED3 has two, mismatched bases (Table 2). The mutation frequency using FnCpf1 with cr*OsNCED1*-1 in rice calli was 2.14–23.3% in the target gene (*OsNCED1*), while those in the off-target genes (*OsNCED2* and *OsNCED3*) were 0–6.25% and 0%, respectively (Table 2). Next, cr*OsAO1-*1 was designed to target both the *OsAO1* and *OsAO2* genes. The cr*OsAO1-*1 has one mismatched base on the corresponding region of the *OsAO3* and *OsAO4* genes, and two mismatched bases in the *OsAO5* gene. When cr*OsAO-1* was used with FnCpf1, mutation frequencies in the *OsAO1, OsAO2,* and *OsAO4* genes were 38.8–50%, 24.1–36.6% and 0–5%, respectively (Table 2). No mutations were observed in *OsAO3* and *OsAO5*.

## Discussion

In this study, we evaluated the use of FnCpf1 in targeted mutagenesis of rice and tobacco genomes; our data showed clearly that FnCpf1 can be applied to targeted mutagenesis in these crops.

Zetsche *et al*. evaluated the genome editing activity of various Cpf1 proteins via transient assay in human cells^13^. Their results revealed that AsCpf1 and LbCpf1 exhibited a higher activity to induce mutation than other Cpf1 enzymes, including FnCpf1^13^. In our study, FnCpf1 was able to effectively induce mutations in various target genes upon constitutive expression of FnCpf1 in tobacco and rice. We previously reported that the engineering of binary vectors expressing SpCas9 significantly affected the mutation ratio in rice^17^. To enhance the expression of FnCpf1 in tobacco and rice, previous studies have introduced a variety of devices such as codon-optimization of FnCpf1, addition of a nuclear localization signal sequence to FnCpf1, translational enhancer, transcriptional terminator and constitutive promoters, to the binary vectors used^17^,^19^. It is highly likely that a combination of a stable expression system and these additional tweaks contributed to improving the genome editing activity of FnCpf1 in plant cells.

In our targeted mutagenesis experiments, the average mutation frequencies on targeted loci in tobacco and rice were 28.2% and 47.2%, respectively. The average mutation frequency in tobacco was lower than that in rice. In addition, we successfully isolated bialellic mutants in the T0 generation in rice but not in tobacco. This may be due to differences in the transformation processes between rice and tobacco. In the case of rice transformation, dedifferentiated callus showing relatively higher cell division activity was utilized for Agrobacterium infection, and the callus state was maintained until the regeneration step^30^. On the other hand, the tobacco transformation process started from leaf discs. Transformed leaf discs were subjected to selection on antibiotics. During this process, screening proceeded in parallel with the other processes, including callus induction and regeneration. When we applied SpCas9 to targeted mutagenesis in rice, mutation ratio and frequency increased in accordance with the duration of the callus state^31^. Therefore, it may be necessary to prolong the duration of the callus state in tobacco transformation in order to isolate biallelic mutants in the T0 generation.

FnCpf1 induced mostly chimeric mutations in tobacco, with various mutations being observed in each regenerated plant (Fig. 2b). On the other hand, in our previous study using SpCas9^17^,^32^, each regenerated rice plant possessed monoallelic or biallelic mutation. This difference may due to the difference in the transformation process as described above. Rice plants could be regenerated mostly from genetically homogeneous callus, and SSN-induced mutation rarely occurs in regenerated rice plants^33^, whereas tobacco might accumulate mutation events in going from regeneration to the reproductive stage. As a result, constitutively occurring mutations could create genetically chimeric plants. When targeted mutagenesis was performed with SpCas9 driven by ubiquitously active promoters, such as 35 S or the ubiquitin promoter, chimeric mutations occurred similarly in tobacco or Arabidopsis^12^,^34^. To address this, several groups have already reported the use of tissue-specific promoters to express SpCas9, e.g., in flower meristem or germ line cells, and have succeeded in reducing chimeric mutation in Arabidopsis^34^,^35^. Therefore, application of promoters functioning in an inducible or tissue-specific manner to control the expression of FnCpf1 spatiotemporally could contribute to reducing chimeric mutation.

FnCpf1-induced mutations were mostly deletions. The mutation patterns of FnCpf1 were similar to those of TALENs, ZFNs and paired nickases (Cas9)^33^,^36^,^37^,^38^. FnCpf1 generates DNA ends with 5′ overhangs; TALENs, ZFNs and paired nickases also generate sticky DNA ends after cleavage of target sequences. It is highly possible that similar DNA repair mechanisms operate after cleavage with these nucleases. DSBs activate DNA repair machinery such as homology dependent repair (HDR) or non-homologous end joining (NHEJ)^39^. DSB repair is affected greatly by the structure of the DNA ends^39^. Cohesive DSBs with compatibility tend to be repaired by precise end joining, while non-compatible DSB ends with various deletions are repaired via NHEJ^40^. In our experiments, we tried to introduce mutations in a single gene with a single crRNA. Single digestion of a target locus generates a DSB with compatibility, and most such DSBs could be repaired precisely.

Four crRNAs were designed for the *NtSTF1* gene, Three of which did not induce mutations in the target loci. Two possible explanations can be considered: (1) there could be epigenomic modification or chromatin structure around the target regions, which could decrease the accessibility of FnCpf1 to the target site^41^,^42^. (2) The secondary structure of FnCpf1 crRNA; FnCpf1 crRNA is 43 nt in length, while the single chimeric guide RNA of Cas9 is around 100 nt^9^,^13^. The crRNA of FnCpf1 consists of two parts, which are responsible for scaffold (19 nt) and target recognition (24 nt), respectively^13^. The interaction between FnCpf1 protein and crRNA could be affected by the secondary structure of the crRNA, which depends strongly on that part of the target sequence. Chemical modification of the crRNA, or an artificially designed crRNA, may improve the interaction between FnCpf1 and crRNA. Chemical modification of the ribonucleotide in the guide RNA of SpCas9 improved mutation frequency by altering RNA stability, and the secondary structure of the guide RNA^43^,^44^. Since FnCpf1 has RNase III activity and trims its crRNA by itself^45^, expression of FnCpf1 crRNA with extra oligonucleotides contributing to preventing unfavorable secondary structure of the crRNA may improve the mutation efficiency of FnCpf 1 as long as a stable transformation system is used to express FnCpf1 with crRNA in plant cells.

Although off-target mutation of FnCpf1 was found in two of five off-target genes in rice (Table 2), mutation frequencies at off-target genes were lower than those at on-target genes (Table 2). Each crRNA had one base mismatch in these two off-target genes, *OsNCED2* and *OsAAO4* (Table 2). A mismatch was found at 11th nucleotide from PAM. We also found an off-target mutation in the *NtSTF1* gene in the S genome of tobacco. The mismatched nucleotide was found just next to the PAM sequence (Fig. 2b,c). Similar to our results, AsCpf1 exhibited consistent tolerance to a single mismatch at positions 1, 8, 9 and 19–23 within the 24 nt spacer sequence in human cells^46^. The problem of off-target mutation by FnCpf1 should be improved by increasing the fidelity of FnCpf1. In the case of SpCas9, the fidelity of target recognition depends greatly on precise recognition of PAM^47^,^48^,^49^; as the PAM sequence becomes longer, fidelity increases. The length of PAM restricts the number of target sites; as PAM becomes shorter, the frequency of occurrence of target sequences in the genome increases. Crystal-structure-based rational engineering of the positively charged groove between the HNH-, RuvC- and PAM-interacting domains in SpCas9 improved the fidelity of target recognition^50^, thus proving it is possible to improve the fidelity of FnCpf1 by structure-based engineering without increasing PAM length.

It was shown recently that AsCpf1 only rarely induces off-target mutation during genome editing in human cells and mice^46^,^51^,^52^. AsCpf1 may have higher fidelity than FnCpf1 since the PAM sequences of FnCpf1 and AsCpf1 are TTN and TTTN, respectively. Application of AsCpf1 and LbCpf1 will be the next challenge in expanding the utility of Cpf1 in plant genome editing.

## Methods

### Vector construction

Two types of FnCpf1 coding sequence were synthesized to optimize codon usage for *Arabidopsis thaliana* and *Oryza sativa*, respectively. The coding sequence of each codon-optimized nuclease, FnCpf1 (At) and FnCpf1 (Os), was cloned into the binary vectors, pRI201-AN (TaKaRa, Japan) and pPZP200^53^, respectively. The crRNA of FnCpf1 was placed under the control of the U6-26 promoter from Arabidopsis, or the U6-2 promoter from rice^17^,^19^; 24 nt target sequences were inserted into the *Bbs* I site next to the crRNA. The expression cassette of FnCpf1 crRNA with target sequences was cloned into the site generated by digestion of the binary vector using two restriction enzymes, *Asc* I and *Pac* I.

### Transformation of tobacco or rice

For tobacco transformation, the binary vector harboring *FnCpf1 (At)* was introduced into *Agrobacterium* strain LBA4404. Leaf discs (8 mm diameter) collected from fully expanded leaves of tobacco (*Nicotiana tabacum* L. cv. Petit Havana SR-1) were used for *Agrobacterium*-mediated transformation as described in Kaya *et al*.^12^. For rice transformation, the *Agrobacterium* strain EHA105 transformed with the binary vector containing *FnCpf1 (Os)*, was used to infect scutellum-derived rice callus (*Oryza sativa* L. ssp. japonica cv. Nipponbare). Details of the rice transformation procedure have been described previously^32^.

### CAPS analysis and heteroduplex mobility assay

Genomic DNA was extracted from regenerated shoots of tobacco, hygromycin-resistant rice calli, or regenerated rice plants, using Agencourt Chloro Pure (BECKMAN COULTER, USA), and target loci were amplified by PCR using the primer sets listed in supplemental table 1. For cleaved amplified polymorphic sequences (CAPS) analysis, PCR products were digested by the appropriate restriction enzymes, and then analyzed by agarose gel electrophoresis. A heteroduplex mobility assay (HMA) was performed using MultiNA (SHIMADZU, Japan) according to our previous report^12^.

### Sequencing analysis

PCR products used in CAPS analysis or HMA were cloned into pCR-BluntII-TOPO (Thermo Fisher Scientific, USA). DNA sequence was determined using a 3500xL genetic analyzer (Applied Biosystems, USA).

## Additional Information

**How to cite this article**: Endo, A. *et al*. Efficient targeted mutagenesis of rice and tobacco genomes using Cpf1 from *Francisella novicida. Sci. Rep.* **6**, 38169; doi: 10.1038/srep38169 (2016).

**Publisher's note:** Springer Nature remains neutral with regard to jurisdictional claims in published maps and institutional affiliations.

## Supplementary Material

Supplemental Information

## Figures and Tables

**Table 1:**
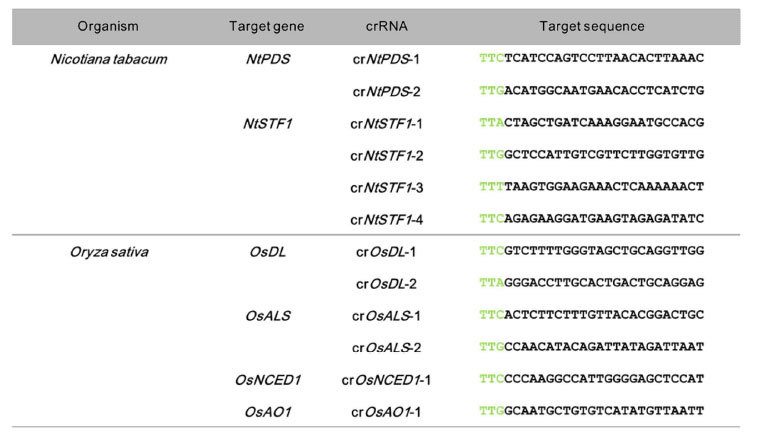
List of target genes, guide RNAs (gRNA), target sequences and PAM sequences used in this study.

Green characters in target sequences indicate PAM motif of FnCpf1.

**Table 2:**
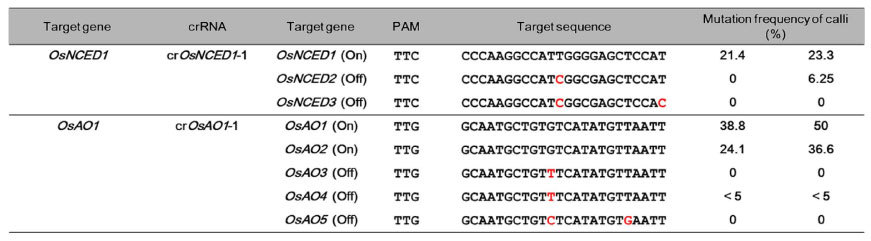
Off-target mutation analysis in *OsNCED* or *OsAAO* gene families in rice.

Red characters indicate mismatched nucleotide of off-target genes against each crRNA.
