# Supplementary material for: Efficient targeted mutagenesis of rice and tobacco genomes using Cpf1 from Francisella novicida
Source: Sci Rep. 2016 Dec 1;6:38169. doi: 10.1038/srep38169 (PMC5131344; doi:10.1038/srep38169)
Supplement: Supplemental Information [file srep38169-s1.pdf]

Supplemental information

Efficient targeted mutagenesis of rice and tobacco genomes using Cpf1 from *Francisella novicida*

Akira Endo[1,+], Mikami Masahumi[1,2,+], Hidetaka Kaya[1] and Seiichi Toki[1,2,3,*]

[1]Plant Genome Engineering Research Unit, Institute of Agrobiological Sciences, National Agriculture and Food Research Organization, 2-1-2 Kannondai, Tsukuba, Ibaraki 305-8602, Japan.

[2]Graduate School of Nanobioscience, Yokohama City University, 22-2 Seto, Yokohama, Kanagawa 236-0027, Japan.

[3]Kihara Institute for Biological Research, Yokohama City University, 641-12 Maioka-cho, Yokohama, Kanagawa 244-0813, Japan.

[+]These authors contributed equally to this work.

[*]Corresponding author: Seiichi Toki

Phone number: +81-29-838-8450

Email address: stoki@affrc.go.jp

Supplemental Figure Legends

Figure S1. Heteroduplex mobility assay of cr*NtSTF1* -1~3 loci.
PCR products were amplified from cr*NtSTF1*-1 (top two panels), cr*NtSTF1*-2 (middle two panels) and cr*NtSTF1*-3 (bottom two panels) loci. Those products were subjected to heteroduplex mobility assay using MultiNA. (S) and (T) indicate PCR products amplified from the loci including each target sequence on *N. sylvestris* and *N. tomentosiformis* genomes, respectively.

Figure S2. *dl* mutant phenotype at T0 generation.
Both plant were transgenic rice possessing *FnCpf1(Os)* with cr*OsDL*-2. *dl/dl* indicates plant having bialellic mutation on cr*OsDL*-2 locus (Left side). *DL/DL* indicates plant without mutation on cr*OsDL*-2 locus (Right side).

Figure S3. CAPS analysis of cr*OsDL*-1 locus in T1 generation of line #18.
-: Non-digested PCR products, +: *Pst* I-digested PCR products. Arrow head indicated the position of undigested PCR products. An undigested band indicates mutation in the target locus.

Figure S4. CAPS analysis of *OsNCED* or *OsAO* gene families to survey off-target mutation.
CAPS analysis of cr*OsNCED1*-1 and cr*OsAO1*-1 loci. -: Non-digested PCR products, + : *Sac* I or *Nde* I-digested PCR products. Arrow head indicated the position of undigested PCR products. An undigested band indicates mutation in the target loci.

Table S1. List of primers used in this study

Plant species	Primer	Sequence	Application
Nicotiana tabacm	NtPDS-1(S) F	ATAGCTGCTGCTGAGATTTGTAAG	cr*NtPDS*-1 HMA
	NtPDS-1(S) R	GCCATACTAAAGAACAACGAAATGCTTACG	cr*NtPDS*-1 HMA
	NtPDS-1(T) F	AAATAGCTGCTGATGAGTTTTGCAAG	cr*NtPDS*-1 HMA
	NtPDS-1(T) R	GGCCATACTAAAGAACAATGAAATGCTTACA	cr*NtPDS*-1 HMA
	NtPDS-2(S&T) F	GGATTCTAACCTGAAGAAATCTGTTCAAAGC	cr*NtPDS*-2 HMA
	NtPDS-2(S&T) R	TGGATGAGAAAGCAAGTGCGTG	cr*NtPDS*-2 HMA
	NtSTF-1(S) F	AGCAGGAGGAGTGGCAGAAT	cr*NtSTF*-1(S) HMA
	NtSTF-1(S) R	CTACTGCATATTATTGGAGAAGTAGTTCCAGAT	cr*NtSTF*-1(S) HMA
	NtSTF-1(T) F	GCAGCAGGAGTGGCAGAATG	cr*NtSTF*-1(T) HMA
	NtSTF-1(T) R	GACTACTACTGCAAATTATTGGAGTAGTAATAG	cr*NtSTF*-1(T) HMA
	NtSTF-2(S) F	CTACTGCAGCAATTAGTAATACTATATCTGGA	cr*NtSTF*-2(S) HMA
	NtSTF-2(S) R	AACAATGTTTTCCATATTTTCGTGTTTACG	cr*NtSTF*-2(S) HMA
	NtSTF-2(T) F	TCATCTACTGCATCAATCAGTAATACTATTACTAC	cr*NtSTF*-2(T) HMA
	NtSTF-2(T) R	AAGATTGTTTTCGATATTTTCGTGTTTGTT	cr*NtSTF*-2(T) HMA
	NtSTF-3(S) F	GCAGCAGCAATGATAACAACGACGATA	cr*NtSTF*-3(S) HMA
	NtSTF-3(S) R	CATACACATGATACAAATGCATTTACG	cr*NtSTF*-3(S) HMA
	NtSTF-3(T) F	GCAGCAGCAACGACGATAATAATC	cr*NtSTF*-3(T) HMA
	NtSTF-3(T) R	CATAGACATGGTCCAAATGCATTTTAAG	cr*NtSTF*-3(T) HMA
	NtSTF-4(S) F	GGCACCCATGATCCATCCTATCAA	cr*NtSTF*-4(S) CAPS
	NtSTF-4(S) R	CATACACATGATACAAATGCATTTACG	cr*NtSTF*-4(S) CAPS
	NtSTF-4(T) F	GGCACCCATGATCCATCCTATCAC	cr*NtSTF*-4(T) CAPS
	NtSTF-4(T) R	CATAGACATGGTCCAAATGCATTTTAAG	cr*NtSTF*-4(T) CAPS
	Tar-Cpf1-crRNA_NtPDS-1F	agatTCATCCAGTCCTTAACACTTAAAC	cloning of crRNA into *Bbs* I site
	Tar-Cpf1-crRNA_NtPDS-1R	aaaaGTTTAAGTGTTAAGGACTGGATGA	cloning of crRNA into *Bbs* I site
	Tar-Cpf1-crRNA_NtPDS-2F	agatACATGGCAATGAACACCTCATCTG	cloning of crRNA into *Bbs* I site
	Tar-Cpf1-crRNA_NtPDS-2R	aaaaCAGATGAGGTGTTCATTGCCATGT	cloning of crRNA into *Bbs* I site
	Tar-Cpf1-crRNA_NtSTF-1F	agatCTAGCTGATCAAAGGAATGCCACG	cloning of crRNA into *Bbs* I site
	Tar-Cpf1-crRNA_NtSTF-1R	aaaaCGTGGCATTCCTTTGATCAGCTAG	cloning of crRNA into *Bbs* I site
	Tar-Cpf1-crRNA_NtSTF-2F	agatGCTCCATTGTCGTTCTTGGTGTTG	cloning of crRNA into *Bbs* I site
	Tar-Cpf1-crRNA_NtSTF-2R	aaaaCAACACCAAGAACGACAATGGAGC	cloning of crRNA into *Bbs* I site
	Tar-Cpf1-crRNA_NtSTF-3F	agatTAAGTGGAAGAAACTCAAAAAACT	cloning of crRNA into *Bbs* I site
	Tar-Cpf1-crRNA_NtSTF-3R	aaaaAGTTTTTTGAGTTTCTTCCACTTA	cloning of crRNA into *Bbs* I site
	Tar-Cpf1-crRNA_NtSTF-4F	agatAGAGAAGGATGAAGTAGAGATATC	cloning of crRNA into *Bbs* I site
	Tar-Cpf1-crRNA_NtSTF-4R	aaaaGATATCTCTACTTCATCCTTCTCT	cloning of crRNA into *Bbs* I site
	AtU6-26 F	ATCTTGTTTCATAGTTTGTCCCAGG	crRNA sequence check
Oryza sativa	OsDL-1F	cagtgtcatgttccatctttcgcttccatt	cr*OsDL*-1 CAPS
	OsDL-1R	atgggcaagagagaaatcttttgcaatcca	cr*OsDL*-1 CAPS
	OsDL-2F	tgcaaaagatttctctcttgcccatctgtg	cr*OsDL*-2 CAPS
	OsDL-2F	tttctcacCTCATGAAGCGGTTGTAAGCAG	cr*OsDL*-2 CAPS
	OsALS-1F	AATTATGCCGTGGATAAGGCTGACCTGTTG	cr*OsALS*-1,2 CAPS
	OsALS-1R	acccaataagatcgaccgaagagagggaaa	cr*OsALS*-1,2 CAPS
	OsNCED1-1F	GTCGGGGAAGAGTGCCATTGGCGTCCCGAA	cr*OsNCED1*-1 CAPS
	OsNCED1-1R	CTTCTTGATCACGTCGTAGCTGAGCGCGTG	cr*OsNCED1*-1 CAPS
	OsNCED2-1F	CGGCGAGGAGGTGTGGCAAGAAGAAGGATG	cr*OsNCED1*-1 CAPS off-target
	OsNCED2-1R	GGCTGCTCAAGCTCGATCTCGACGTCGGGT	cr*OsNCED1*-1 CAPS off-target
	OsNCED3-1F	GCAACCGTGCCAACCAATGGCAAGATCAAG	cr*OsNCED1*-1 CAPS off-target
	OsNCED3-1R	CCGTCGGCGGTGAAGTAGAAGTACTTGAGG	cr*OsNCED1*-1 CAPS off-target
	OsAAO1-1F	ACATCATCATGGCGCAGAGGTTGTCTTTCT	cr*OsAAO1*-1 CAPS
	OsAAO1-1R	AGAGAATATGTACCAGAAGCTTGGAGCTCA	cr*OsAAO1*-1 CAPS
	OsAAO2-1F	ACATCATCATGGCGCAGAGGCTGTCTTTCC	cr*OsAAO1*-1 CAPS
	OsAAO2-1R	TCTAAGATGCTCTAACATGGGTAGCACGAA	cr*OsAAO1*-1 CAPS
	OsAAO3+4-1F	GGCACACAAATACCCTTTTCGCTCAGACAT	cr*OsAAO1*-1 CAPS off-target
	OsAAO3-1R	TATCCTTGGCAGAAACAACTGTAAGGATCTTCTTT	cr*OsAAO1*-1 CAPS off-target
	OsAAO4-1R	ACCACAACACCGAGAACTTGTCCAGCAAAC	cr*OsAAO1*-1 CAPS off-target
	OsAAO5-1F	CAAAGCCATCGAGATACTACGGTCAGATGG	cr*OsAAO1*-1 CAPS off-target
	OsAAO5-1R	CGACTTTCTTGATTGGTTTTCCGACTGGTT	cr*OsAAO1*-1 CAPS off-target
	Tar-Cpf1-crRNA_DL-1F	AGATgtcttttgggtagCTGCAGGTTGG	cloning of crRNA into *Bbs* I site
	Tar-Cpf1-crRNA_DL-1R	AAAACCAACCTGCAGctacccaaaagac	cloning of crRNA into *Bbs* I site
	Tar-Cpf1-crRNA_DL-2F	AGATgGGACCTTGCACTGACTGCAGGAG	cloning of crRNA into *Bbs* I site
	Tar-Cpf1-crRNA_DL-2R	AAAACTCCTGCAGTCAGTGCAAGGTCCc	cloning of crRNA into *Bbs* I site
	Tar-Cpf1-crRNA_ALS-2F	AGATccaacatacagattatagaTTAAT	cloning of crRNA into *Bbs* I site
	Tar-Cpf1-crRNA_ALS-2R	AAAAATTAAtctataatctgtatgttgg	cloning of crRNA into *Bbs* I site
	Tar-Cpf1-crRNA_ALS-1F	AGATACTCTTCTTTGTTACACGGACTG	cloning of crRNA into *Bbs* I site
	Tar-Cpf1-crRNA_ALS-1R	AAAACAGTCCGTGTAACAAAGAAGAGT	cloning of crRNA into *Bbs* I site
	Tar-Cpf1-crRNA_NCED1-1F	AGATCCCAAGGCCATTGGGGAGCTCCAT	cloning of crRNA into *Bbs* I site
	Tar-Cpf1-crRNA_NCED1-1R	AAAAATGGAGCTCCCCAATGGCCTTGGG	cloning of crRNA into *Bbs* I site
	Tar-Cpf1-crRNA_AAO-1F	AGATGCAATGCTGTGTCATATGTTAATT	cloning of crRNA into *Bbs* I site
	Tar-Cpf1-crRNA_AAO-1R	AAAAAATTAACATATGACACAGCATTGC	cloning of crRNA into *Bbs* I site
	OsU6-2-F	TGCTGGAATTGCCCTTGGATCATGAACCAA	crRNA sequence check

Figure S1.

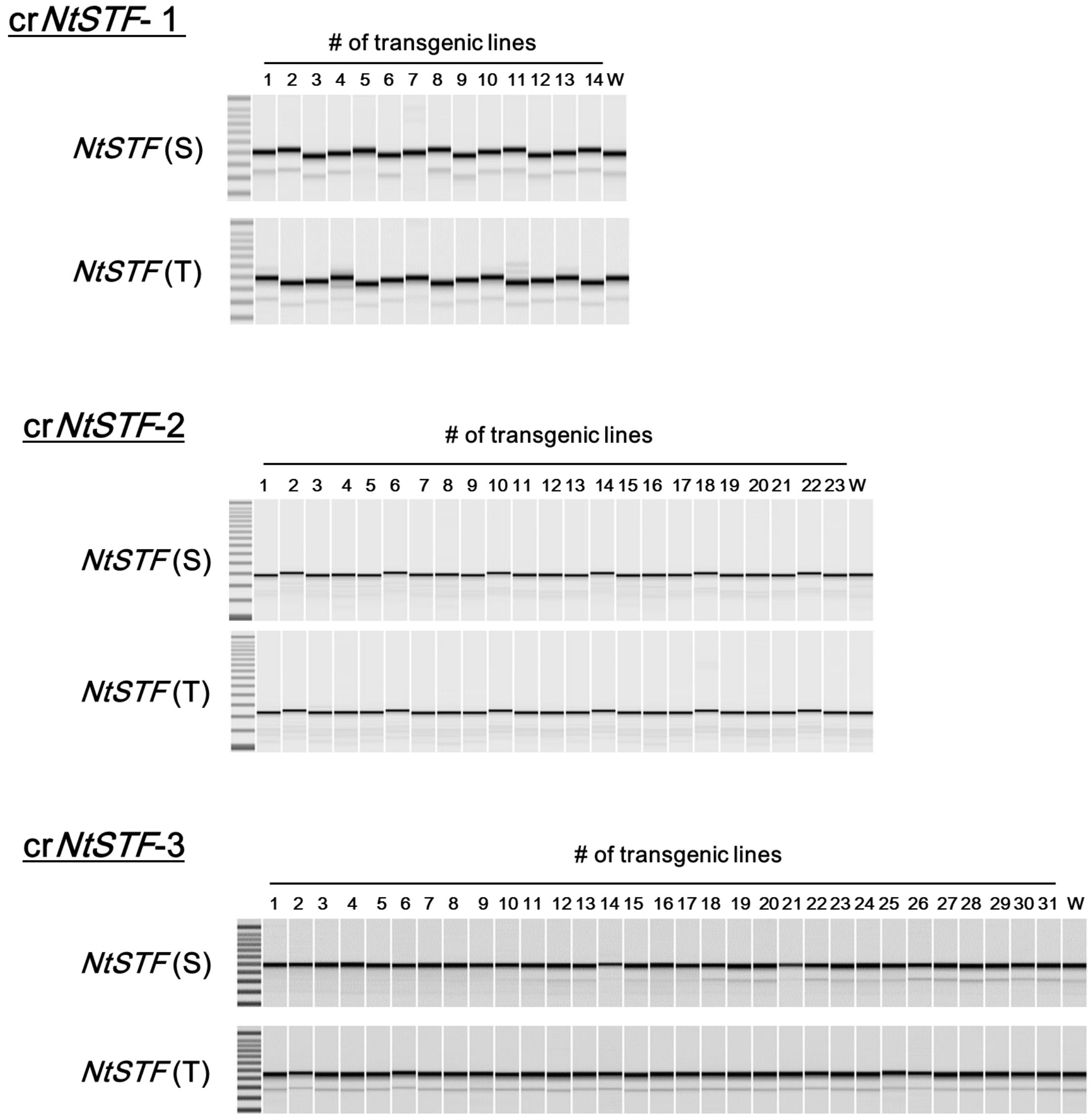
crNtSTF- 1
# of transgenic lines
1 2 3 4 5 6 7 8 9 10 11 12 13 14 W
NtSTF (S)
NtSTF (T)
crNtSTF-2
# of transgenic lines
1 2 3 4 5 6 7 8 9 10 11 12 13 14 15 16 17 18 19 20 21 22 23 W
NtSTF (S)
NtSTF (T)
crNtSTF-3
# of transgenic lines
1 2 3 4 5 6 7 8 9 10 11 12 13 14 15 16 17 18 19 20 21 22 23 24 25 26 27 28 29 30 31 W
NtSTF (S)
NtSTF (T)

Figure S2.

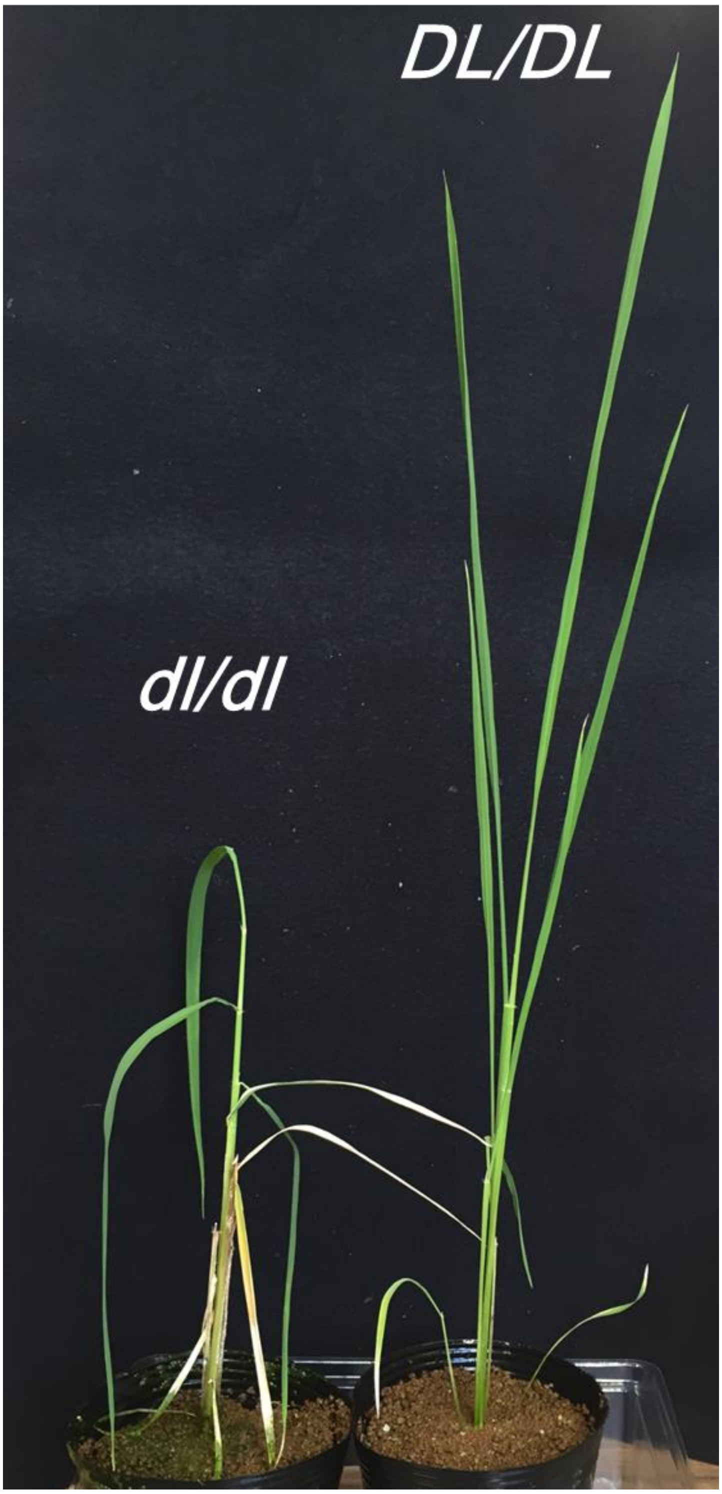

Figure S3.

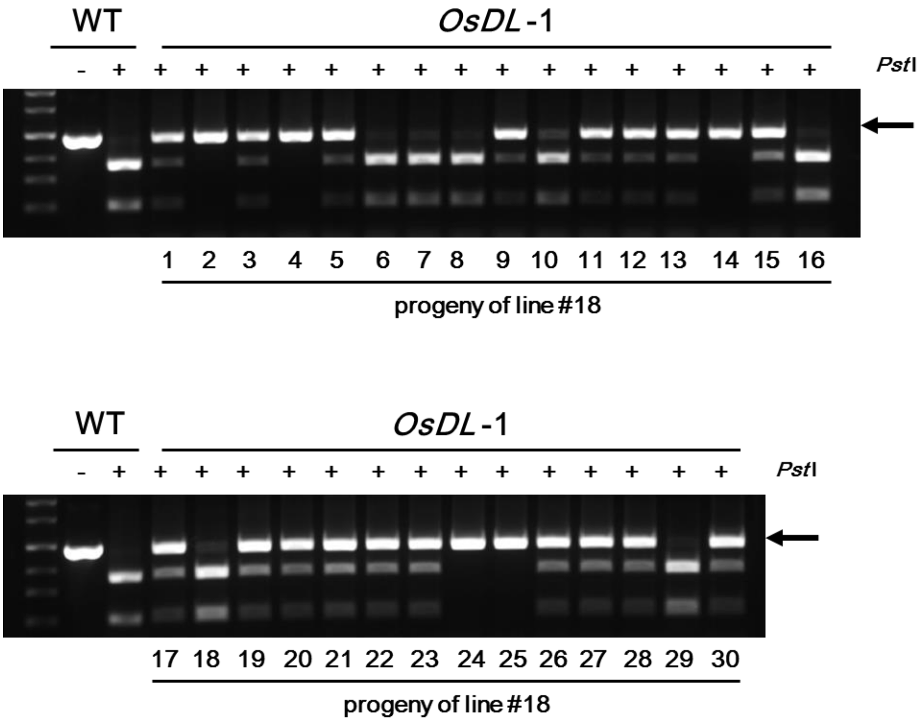
WT
OsDL-1
- + + + + + + + + + + + + + + + + +
PstI
1 2 3 4 5 6 7 8 9 10 11 12 13 14 15 16
progeny of line #18
WT
OsDL-1
- + + + + + + + + + + + + + + +
PstI
17 18 19 20 21 22 23 24 25 26 27 28 29 30
progeny of line #18

Figure.S4

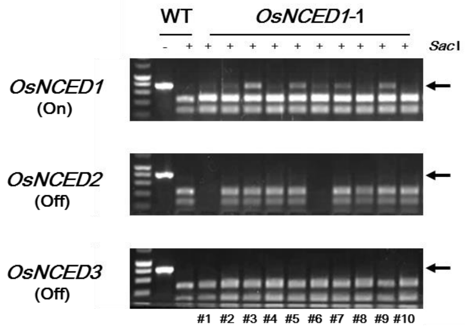
WT
OsNCED1-1
SacI
OsNCED1
(On)
OsNCED2
(Off)
OsNCED3
(Off)
#1 #2 #3 #4 #5 #6 #7 #8 #9 #10

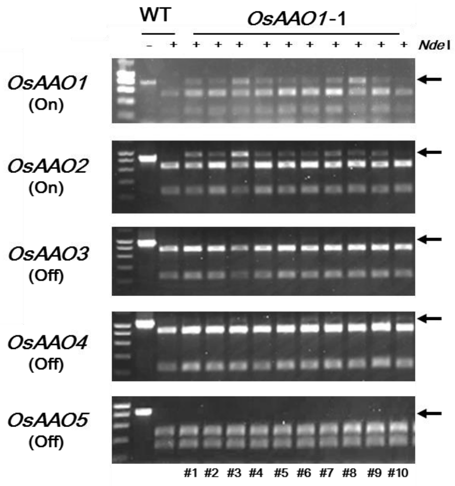
WT
OsAAO1-1
NdeI
OsAAO1
(On)
OsAAO2
(On)
OsAAO3
(Off)
OsAAO4
(Off)
OsAAO5
(Off)
#1 #2 #3 #4 #5 #6 #7 #8 #9 #10
